# Co-expression analysis of lncRNA and mRNA identifies potential adipogenesis regulatory non-coding RNAs involved in the transgenerational effects of tributyltin

**DOI:** 10.1371/journal.pone.0281240

**Published:** 2023-02-03

**Authors:** Maria Fernanda da Silva Lopes, Juliana de Souza Felix, Natália Francisco Scaramele, Mariana Cordeiro Almeida, Amanda de Oliveira Furlan, Jéssica Antonini Troiano, Flávia Regina Florêncio de Athayde, Flávia Lombardi Lopes

**Affiliations:** 1 Department of Animal Production and Health, School of Veterinary Medicine, São Paulo State University Júlio de Mesquita Filho (Unesp), Araçatuba, Brazil; 2 Faculdades de Dracena (UNIFADRA–Fundec), Dracena, São Paulo, Brazil; Massachusetts General Hospital, UNITED STATES

## Abstract

The obesity epidemic is considered a global public health crisis, with an increase in caloric intake, sedentary lifestyles and/or genetic predispositions as contributing factors. Although the positive energy balance is one of the most significant causes of obesity, recent research has linked early exposure to Endocrine-Disrupting Chemicals (EDCs) such as the obesogen tributyltin (TBT) to the disease epidemic. In addition to their actions on the hormonal profile, EDCs can induce long-term changes in gene expression, possibly due to changes in epigenetic patterns. Long non-coding RNAs (lncRNAs) are epigenetic mediators that play important regulatory roles in several biological processes, through regulation of gene transcription and/or translation. In this study, we explored the differential expression of lncRNAs in gonadal white adipose tissue samples from adult male C57BL/6J F4 generation, female C57BL/6J offspring exposed (F0 generation) to 50 nM TBT or 0.1% DMSO (control of vehicle) via drinking water provided during pregnancy and lactation, analyzing RNA-seq data from a publicly available dataset (GSE105051). A total of 74 lncRNAs were differentially expressed (DE), 22 were up-regulated and 52 were down-regulated in the group whose F4 ancestor was exposed in utero to 50nM TBT when compared to those exposed to 0.1% DMSO (control). Regulation of DE lncRNAs and their potential partner genes in gonadal white adipose tissue of mice ancestrally exposed to EDC TBT may be related to the control of adipogenesis, as pathway enrichment analyses showed that these gene partners are mainly involved in the metabolism of lipids and glucose and in insulin-related pathways, which are essential for obesity onset and control.

## Introduction

Exposure to environmental factors during embryonic development has been linked to increased risk of diseases such as obesity and type 2 diabetes mellitus later in life. The obesity epidemic is considered a global public health crisis, having as contributing factors increased caloric intake, sedentary lifestyles and/or genetic predispositions. Although the positive energy balance is one of the most significant causes of obesity, recent research has linked early exposure to endocrine disrupting chemicals (EDCs) to the disease [1].

EDCs are chemical compounds that interfere with production, release, transport, metabolism, action or elimination of endogenous hormones responsible for maintaining homeostasis and regulating developmental processes [2]. Obesogenic substances comprise a subset of EDCs, which can lead to accumulation of lipids through inadequate adipogenesis, hypertrophy or hyperplasia of adipocytes, or by affecting hormonal regulation of metabolism, appetite and satiety [3]. There is growing evidence suggesting that exposure to these chemicals during intrauterine development or lactation can strongly influence the offspring’s predisposition to obesity in adulthood [4].

The germline transmission of epigenetic information between generations in the absence of direct exposures to environmental factors is defined as transgenerational epigenetic inheritance. Exposure of the pregnant mother (F0), linked with the developing fetus (F1), to environmental insults (e.g. endocrine disruptors, toxics, malnutrition), causes epimutations that are transmitted to the F2 and F3 generation. The transgenerational epigenetic inheritance caused by the environment has significant consequences in the etiology of diseases, inheritance of phenotypic variation and in evolutionary biology [5]. Studies show that transgenerational effects obtained through exposure to environmental factors is associated with epimutations in DNA methylation patterns and in histone retention patterns, which are promoted specifically through the germline [6–8].

Transgenerational effects have been observed with some types of EDCs, such as bisphenol A (BPA), dichlorodiphenyltrichloroethane (DDT), dibutyl phthalate (DBP), triphenyltin (TPT) and tributyltin (TBT). Egusquiza and Blumberg [9] reported that TBT induces obesity by promoting the differentiation of adipocytes in the body while stimulating the activity of the RXR-PPARγ complex, and that the obesogenic effects of TBT exposure are propagated transgenerationally to unexposed offspring through epigenetic changes. Shoucri et al. [10] have demonstrated that exposure to TBT in mesenchymal stem cell culture is related to greater accumulation of lipids during subsequent adipose differentiation. In addition to their actions on the hormonal profile, EDCs can induce long-term changes in gene expression, possibly due to changes in epigenetic patterns [11]. Epigenetic mechanisms play essential roles in the processes that determine adult phenotypes through epigenetic programming. Numerous studies over the last two decades have shown that maternal nutrition can cause changes in the fetal epigenome, i.e., DNA methylation profile, post-translational histone modifications, and regulation of and by non-coding RNAs (ncRNAs), which can lead to permanent phenotypic changes in the offspring, as reviewed by Greco et al. [12].

LncRNAs are non-coding transcripts composed of more than 200 nucleotides, and play an important role in the transcriptional, post-transcriptional and epigenetic regulation of gene expression, thus being able to silence or activate specific genes or loci [13]. The mechanisms by which lncRNAs regulate their targets genes depend on specific features of primary sequence, secondary structure and genomic positioning of lncRNA transcripts. LncRNAs can act by recruiting different protein components of the chromatin remodeling complex to change chromatin organizational patterns; they can function as ’sponges’ by base pairing with complementary miRNAs, thus reducing their effects; they can play scaffolding roles by providing docking sites for proteins that function together in the same biological pathway; lncRNAs can activate transcription of certain genes by guiding transcription factors to their promoters, or suppress transcription by sequestering transcription factors; and they can also modulate mRNA by base pairing with them to inhibit translation, alter splicing patterns or affect degradation [14].

Considering the importance of maternal nutrition for epigenetic patterning on the offspring (and transgenerationally on their descendants), the diverse roles that lncRNAs play on gene expression control, and the previously demonstrated effects of TBT on the expression of genes relevant to fat metabolism [1], we aimed to evaluate the effects of ancestral exposure to obesogenic substances on the expression of lncRNAs, and to correlate their expression to those of their possible biological targets in the white adipose tissue (WAT) of mice.

In this study, we identified lncRNA expression profiles in the WAT of F4 mice transgenerationally exposed to 50nM TBT or 0.1% DMSO (control). Differentially expressed lncRNAs were then used to predict putative *cis*- and *trans*-target genes which were then integrated with differentially expressed mRNA data to improve the accuracy of the target prediction. Putative target mRNAs of lncRNAs in *cis* and *trans* were then used to build lncRNA-mRNA correlation networks affected transgenerationally in F4, following exposure of F0 generation to the obesogen TBT.

## Methods

### RNA-seq datasets

RNA-seq data were previously generated by Chamorro-Garcia et al. [1], and obtained from the Gene Expression Omnibus (GEO) public database (www.ncbi.nlm.nih.gov/geo/) under the bioproject PRJNA414476, with accession number GSE105051. Briefly, 7 week-old female C57BL/6 J mice (generation F0) were exposed to 50 nM TBT or 0.1% DMSO (vehicle control) via drinking water provided during pregnancy and lactation. To form subsequent generations (F2-F4), non-sibling mice were randomly assigned from litters within the same experimental groups. Only animals from the F0 generation were directly exposed to 50 nM TBT (exposed to the TBT) or 0.1% DMSO (control).

Mice were kept on low-fat chow (standard diet, SD—13.2% KCal of fat) throughout the experimentation period (F0-F4). To assess interaction between TBT exposure and dietary fat levels, F4 descendants (n = 4 for each experimental group) of TBT or DMSO F0 females were switched to a high-fat diet (HFD—21.2% KCal of fat) at week 19. These F4 animals were kept in the HFD for 6 weeks, then returned to SD for 8 weeks until 33 weeks of age. To assess the effect of ancestral exposure to TBT on fat mobilization, one week before euthanasia (week 32), animals were submitted to overnight fasting (16h). In total, 8 samples of gonadal WAT (gWAT) were used for RNA-Seq, consisting of 4 samples from the 50 nM TBT exposed group (exposed to TBT in the F0 generation) and 4 samples from the 0.1% DMSO group (not exposed to TBT in the F0 generation).

### Bioinformatic identification of lncRNAs in RNA-seq datasets

Quality of the extracted RNA-seq readings was evaluated with FastQC available at the public server www.usegalaxy.org [15]. Data were aligned to the latest mouse genome reference sequence (GRCm38.p6, as provided by GENCODE) (https://www.gencodegenes.org/) using HISAT2 version 2.1.0+galaxy5 [16] with the Burrows-Wheeler Transformation (BWT) and the Ferragina-Manzini (FM) indexing algorithms. The resulting BAM file was then processed with FeatureCounts version 1.6.4+galaxy1 [17] to perform read counts using the GENCODE M25 (mouse) annotation as reference (https://www.gencodegenes.org/). Quality control of all steps was carried out using MultiQC. Next, DESeq2 (version 2.11.40.6 + galaxy1) was used to perform statistical analyses of differential expression of lncRNAs and mRNAs between samples from the TBT and control (DMSO) groups. This tool estimates the average variance in read counts and tests the differential expression using a binomial distribution model as basis and Wald test [18].

The Biomart tool was used to classify the transcripts according to their biotype. Biotypes are classified according to HAVANA gene biotype (http://www.ensembl.org/info/genome/genebuild/biotypes.html) and grouped into 3 classes: protein-coding genes, long non-coding RNA (lncRNAs) genes and small non-coding RNA genes. In the lncRNAs class, the following descriptions were considered: "processed_transcript", "pseudogene", "To be Experimentally Confirmed (TEC)", "lincRNA", "3prime_overlapping_ncrna", "antisense", "sense_intronic" and sense_overlapping".

Differently expressed (DE) LncRNAs (p < 0.05) and coding proteins (p < 0.05 and log2(FC) ≥ ± 0.5) were clustered with the heatmap3 package in R using the “complete linkage” method and the Euclidean distance as parameters (https://www.rdocumentation.org/packages/heatmap3/versions/1.1.7/topics/heatmap3).

### Prediction analysis of putative lncRNAs with *cis*-and *trans* action

Differentially expressed lncRNAs were used for the prediction of the putative *cis-* and *trans-*target genes. First, using normalized counts of differentially expressed lncRNAs and mRNAs, we performed correlation analysis by means of Pearson’s correlation coefficient. A lncRNA-mRNA interaction was considered significant when Pearson`s correlation |r| ≥ 0.80 and p<0.05. From the total correlation matrix, we performed two analyses to identify and classify the interactions and possible actions of lncRNAs (*cis* and/or *trans*) in relation to their target gene. To check potential lncRNA-mRNA interactions, LncTar (http://www.cuilab.cn/lnctar) [19] was used to predict lncRNA targets (normalized dG (ndG) was set to -0.10) and those with significant correlation, as identified by Pearson’s correlation (as described above), were maintained. Classification of DE lncRNAs was performed using the program FEELnc (Flexible Extraction of Long non-coding RNAs) (v.0.1.1) (https://github.com/tderrien/FEELnc) [20]. Based on locus analysis, lncRNA-mRNA interactions that had the target mRNA within a window of 100 kbps upstream or downstream of the lncRNA location were classified as cis-acting, and interactions outside the established window of 100 kbps and that also had a binding potential (ndG≤ -0.10) were classified as trans-acting.

Only DE lncRNAs with correlation and binding potential within the parameters and their corresponding *cis-* and *trans*-target genes were used to construct lncRNA-gene interaction networks using the Cytoscape 3.9.0 program (https://cytoscape.org/). Also, Cytoscape was used to identify nodes from the co-expression modules. The top five nodes were ranked according to interaction number of the lncRNAs and their targets.

To gather as much information as possible on all lncRNAs, we performed orthology analysis of all DE lncRNAs with humans using the Orthology Predictions Search tool, available at https://www.genenames.org/tools/hcop/#!/. Subsequently, the NcPath tool (http://ncpath.pianlab.cn/#/Home) was employed to compare the predict targets of the orthologous lncRNA to experimentally-verified lncRNA targets in humans.

### Gene ontology and pathway enrichment analysis

We used the g:GOSt (Function Profiling) tool within gProfiler (https://biit.cs.ut.ee/gprofiler/gost) for analysis of the Gene Ontology (GO) annotation and Kyoto Encyclopedia of Genes and Genomes (KEGG Pathways) of all significant lncRNAs and partner mRNA pairs (https://biit.cs.ut.ee/gprofiler/gost). All p-values were adjusted using the Benjamini-Hochberg (FDR) method (adjusted p-value <0.05).

**Fig 1:**
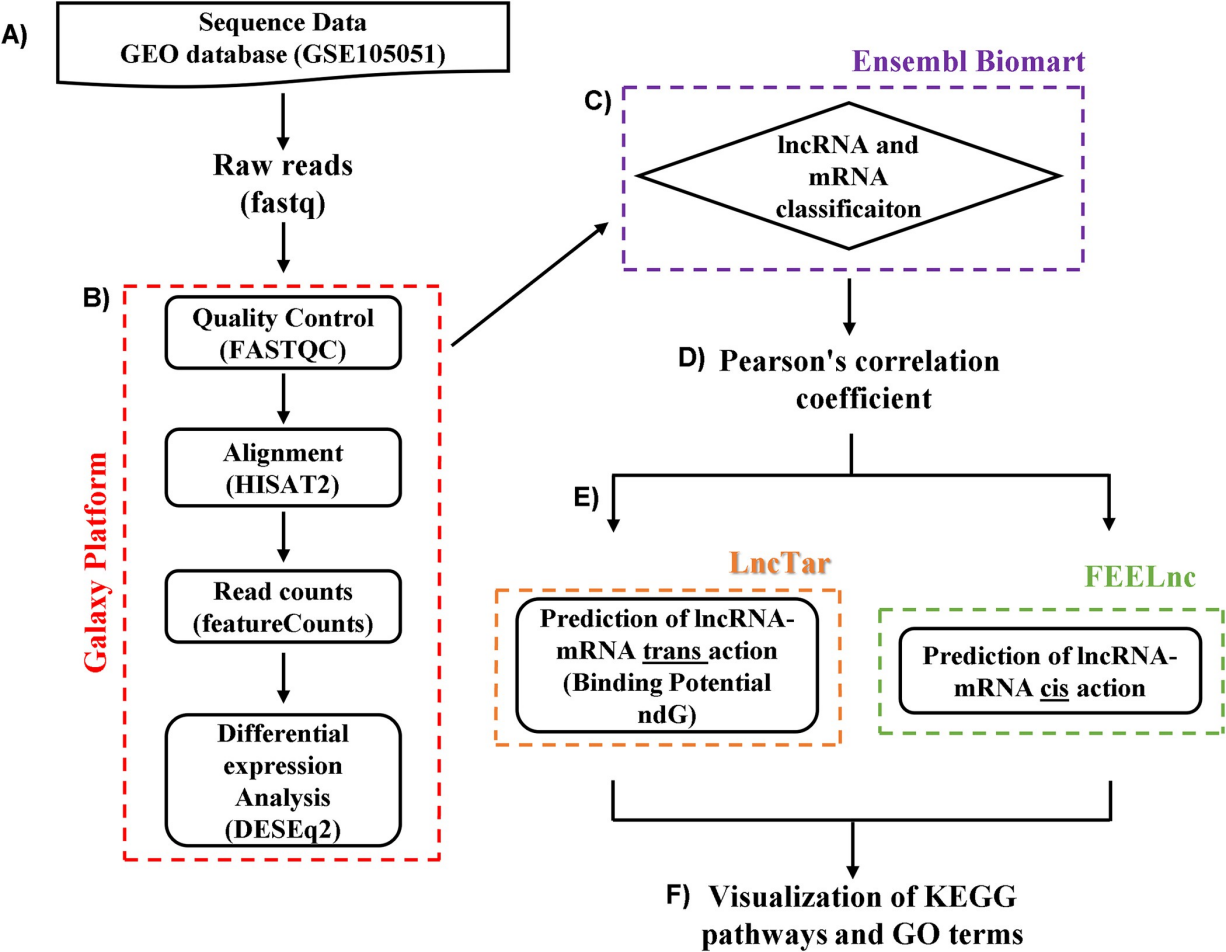
Workflow of lncRNA analysis and functional predictions. The workflow describes the step-by-step bioinformatics analyzes performed in our study. (A) Raw reads were extracted from the GEO database. (B) The bioinformatics analyzes (quality, alignment, counting and differential expression) of these readings were performed on the Galaxy platform. (C) Classification of transcripts was performed on the Ensembl Biomart platform. (D) Correlation between differentially expressed lncRNAs-mRNAs. (E) Classification of the action of lncRNAs and their target mRNAs. (F) Functional enrichment analysis.

A schematic overview of the lncRNA analysis and identification pipeline can be seen in Fig 1.

### MBD-seq data analysis

To evaluate the methylation profile of the regions of interest (DE lncRNAs), MBD-seq data, generated with the same model described for RNA-Seq (Chamorro-Garcia et al. [1]) and previously analyzed by the authors, was employed (GSE105051).

## Results

### Identification differential expression of lncRNA

Out of 818 differentially expressed transcripts, 708 (86.5%) matched to protein coding and 74 (~9%) were considered to have a lncRNA biotype, according to the HAVANA gene biotype classification, available on the Ensembl Biomart tool. Long intergenic non-coding RNAs (lincRNAs) accounted for 39.2% of all DE lncRNAs, followed by antisense transcripts (27%). The remaining non-coding transcript types were TEC (18.9%), processed_transcripts (8.1%), bidirectional promoter lncRNA (5.4%), and sense_intronic transcripts (1.4%).

**Fig 2:**
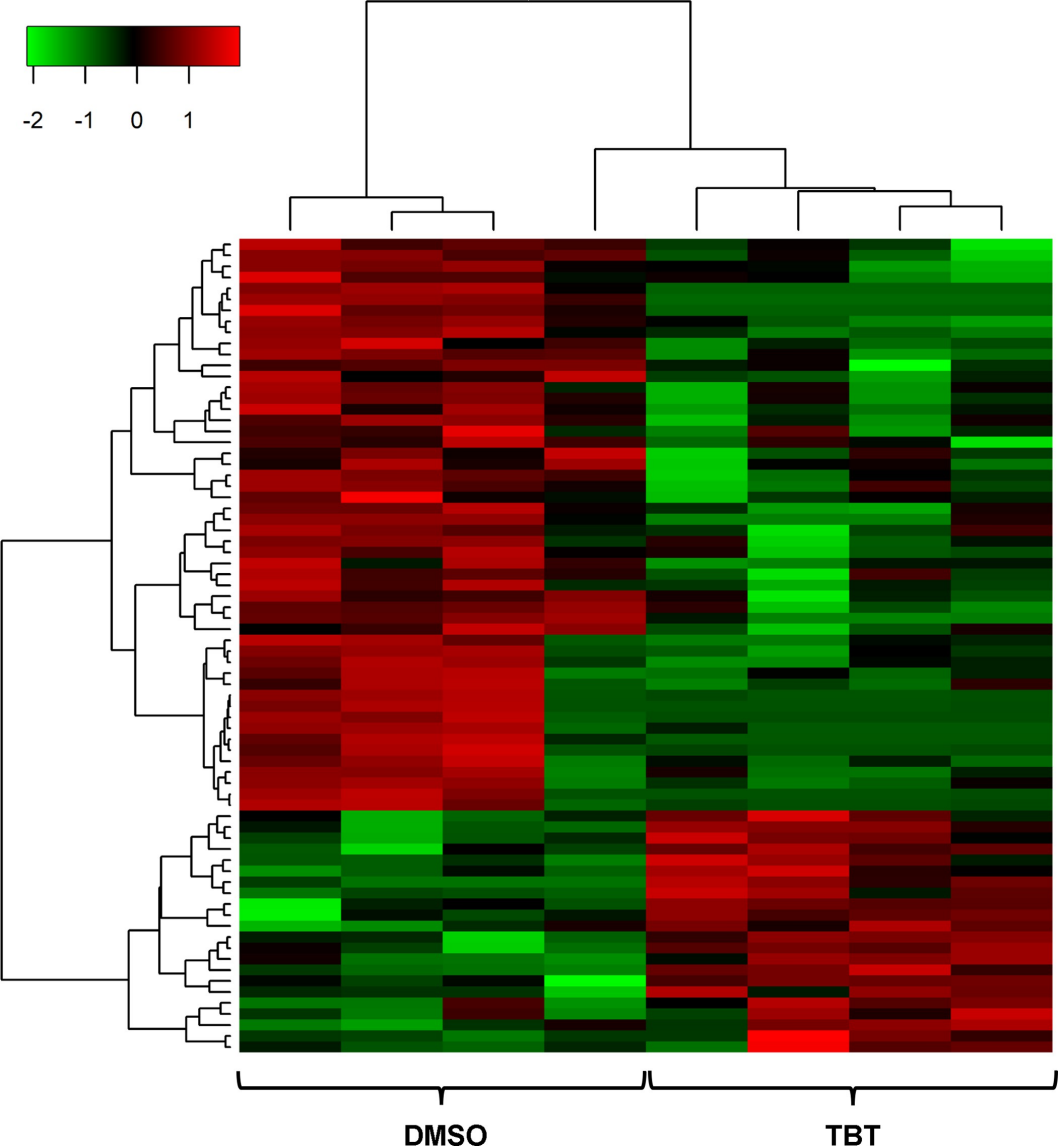
Heatmap of 74 differentially expressed lncRNAs between samples of gWAT ancestrally exposed to EDC TBT and samples of the control group (DMSO). Expression of lncRNA is represented according to the color scale shown at the top, corresponding to the z-score. Red represents higher expression, and green represents lower expression.

Of these 74 DE lncRNAs in the contrast of the groups exposed to 50 nM TBT or exposed to 0.1% DMSO (S1A and S1B Table), 22 showed increased expression in the group in which F0 was exposed to 50 nM TBT when compared to that exposed to 0.1%DMSO. The other 52 lncRNAs were downregulated in the group ancestrally (F4) exposed to 50 nM TBT (Fig 2).

### Correlation of expression and classification of *trans* and *cis* acting lncRNAs

To assess the influence of lncRNAs on the expression of mRNAs and their biological functions, we obtained a correlation matrix of 6595 significant interactions between DE lncRNA and mRNA (Pearson correlation r ≥ |0.80|; p-value <0.05) (S2 Table). Of these, we observed that a total of 1191 lncRNA-mRNA interactions presented significant binding potential, suggesting trans-action potential (normalized deltaG analysis in LncTar tool (ndG < -0.10)) (S3 Table).

In the prediction analysis of *cis* lncRNAs-mRNAs pairs (performed in the FEELnc tool), we obtained 11 DE lncRNAs linked to 11 genes close to DE, and of these 2 lncRNA-mRNA interactions showed significant correlation (Pearson correlation r ≥ |0.80 |; p-value <0.05) (S4 Table). As a cis-acting transcript, lncRNA Gm26704 could affect Fzd6 pre-transcriptionally, in addition to presenting binding potential (LncTar) with the Fzd6 mRNA. The lncRNA Gm10603 is a cis-partner of the *Ucp* gene.

Following orthology analysis of our 74 differentially expressed lncRNAs, only 2 lncRNAs had human orthologous, namely Rian (MEG8—human) and Ftx (FTX—human). Of these, Rian was the only one with predicted targets in mice that were also experimentally-verified targets in humans (Shank2 and Inhba).

### Biological function analysis

**Fig 3:**
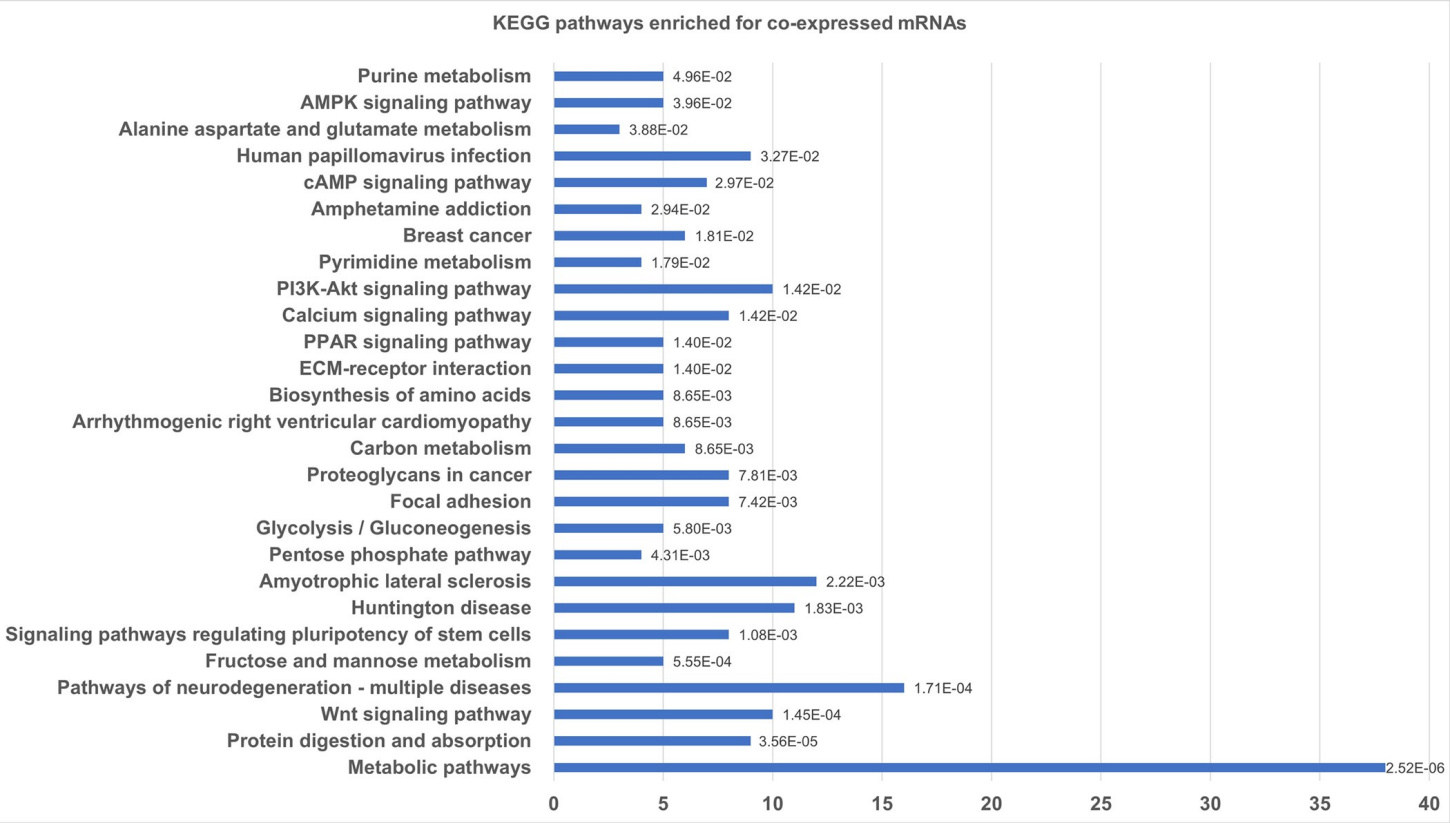
KEGG pathways enriched for co-expressed mRNAs in Cytoscape network. KEGG pathway enrichment analysis was performed in gProfiler. The y-axis represents the KEGG pathways and the x-axis represents the number of genes participating in each pathway. The numbers in front of the bars represent the adjusted p-value of the respective route.

For gene ontology and KEGG pathway analyses, DE mRNAs and lncRNAs that showed significant correlation (Pearson correlation r ≥ |0.80 |; p-value <0.05) and binding potential (ndG < -0.10) were used. Therefore, 479 target genes identified from the 61 lncRNAs were used to obtain information about biological functions. KEGG analysis revealed 27 pathways (Fig 3) with 100 of our identified partner mRNAs, which in turn were potentially regulated by 50 DE lncRNAs. The enriched pathways were mainly related to lipid and glucose metabolism.

**Fig 4:**
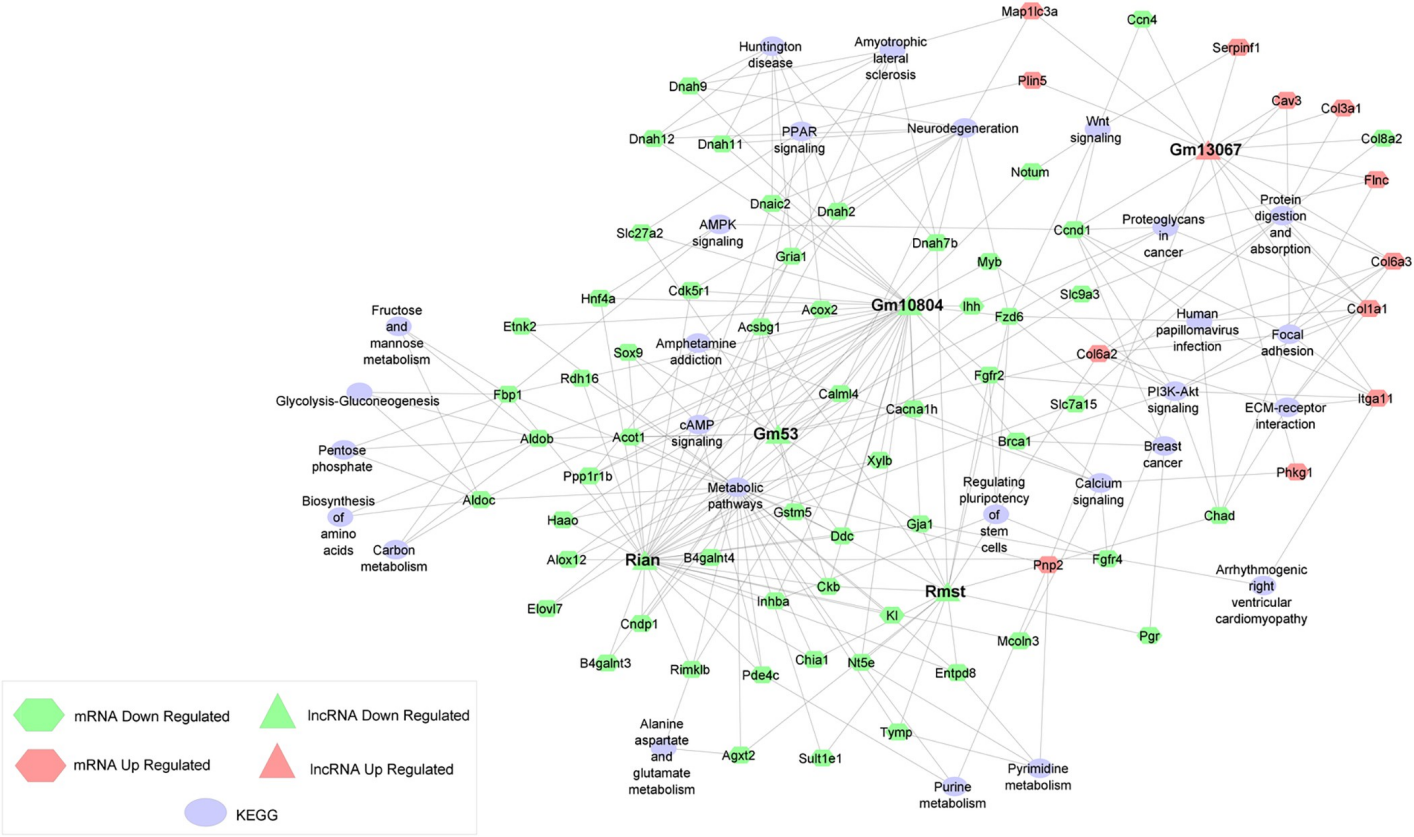
Network analysis of the top 5 network analysis of the top 5 lncRNAs based on the number of interactions with mRNAs. Hexagons represent mRNAs, and triangles represent lncRNAs. Light blue nodes represent KEGG pathways. Expression of lncRNA and mRNA are represented according to the colors red and green, corresponding to up and down-regulated transcripts, respectively.

Next, we constructed co-expression networks to better visualize the five major interactions between lncRNAs and their targets (Fig 4). Using the analyze network tool in Cytoscape, the top five lncRNAs were identified based on the number of interactions with mRNA partners (Table 1 and S5 Table).

**Table 1:** Top 5 lncRNAs based on the number of interactions with mRNAs.

Name	Interactions
Rian	33
Gm10804	32
Rmst	16
Gm13067	14
Gm53	13

### Identification of DE lncRNAs in DMRs

Next, we used the MBD-Seq data obtained by Chamorro-Garcia et al. [1] in order to verify the location of Differentially Methylated Regions (DMR) in relation to our identified DE lncRNAs. In the study, the authors classified the regions according to the distance of the DMRs from the transcription start site (TSS), as well the number of DMRs present. Region I was comprised of genes with at least one DMR in close proximity (between -1500 bp and +500 bp) to the transcription start site (TSS). Region II indicates genes that overlap or flank at least one DMR, regardless of their distance from the TSS. Finally, region III represents genes located in iso-differentially methylated blocks (isoDMBs). Our results show that 35 of our DE lncRNAs were located within regions II and III, as classified by Chamorro-Garcia et al. [1], 9 of which were down-regulated in hypermethylated regions and 10 were up-regulated in hypomethylated regions (Table 2) suggesting that global changes in DNA methylation, resulting from ancestral exposure to the endocrine disruptor TBT, can alter the expression of lncRNAs and mRNAs involved in the adipogenesis process.

**Table 2:** LncRNAs associated to genomic structures defined using TBT-dependent DNA methylome.

Gene symbol	Up/Down	Direction of change	DMR subset	DMR structure
1700018A04Rik	down	Hypermethylated	II	mDMR
2810013P06Rik	down	Hypermethylated	III	isoDMB
3300002A11Rik	down	Hypermethylated	III	isoDMB
Arhgap27os1	down	Hypermethylated	III	isoDMB
B130024G19Rik	down	Hypermethylated	II	mDMR
BE692007	down	Hypermethylated	III	isoDMB
E430024I08Rik	down	Hypermethylated	III	isoDMB
Gm10804	down	Hypermethylated	II	mDMR
Gm37464	down	Hypermethylated	II	mDMR
1700047G03Rik	up	Hypomethylated	III	isoDMB
B430119L08Rik	up	Hypomethylated	II	mDMR
Gm10370	up	Hypomethylated	II	mDMR
Gm10603	up	Hypomethylated	III	isoDMB
Gm13067	up	Hypomethylated	II	mDMR
Gm13375	up	Hypomethylated	II	mDMR
Gm42917	up	Hypomethylated	III	isoDMB
Gm43050	up	Hypomethylated	III	isoDMB
Gm5144	up	Hypomethylated	III	isoDMB
Gm5627	up	Hypomethylated	III	isoDMB

## Discussion

Exposure to endocrine-disrupting chemicals has been linked to transgenerational effects, including a predisposition to unfavorable phenotypic traits and the development of diseases, including obesity and other associated comorbidities [21]. In the present study, we analyzed the expression profiles of lncRNAs in the gWAT of F4 mice ancestrally exposed in F0 to obesogenic substances. Previous evidence indicates that epigenetic mechanisms, e.g. DNA methylation, histone methylation, histone retention and the expression of non-coding RNAs may be involved in transgenerational inheritance under the effects of endocrine disruptors [1, 22]. With a particular interest in ncRNA regulation of phenotypes, we used the RNA-Seq data elegantly generated by Chamorro-Garcia et al. [1], and identified a total of 74 differentially expressed lncRNAs in F4 mice ancestrally exposed in F0 to the EDC TBT, and analyzed the expression correlation with their presumptive partner genes, as lncRNAs are known to regulate protein-coding genes [23]. Among the many regulatory roles of lncRNAs [24], the use of transcriptome data affords the investigation of direct effects of lncRNAs on coding transcripts, through the use of target sequence-based prediction and coexpression analyses. In order to investigate the biological functions of these lncRNAs and their gene partners, we performed GO term analysis and pathway enrichment analysis. Pathway analysis showed that some of our DE lncRNAs and their partner genes are primarily involved in glucose and lipid metabolism and in insulin-related pathways, essential in regulating adipogenesis and obesity [25].

LncRNA Gm6277, upregulated in the TBT group, is correlated with the coding transcript for *Slc2a4* (Solute Carrier Family 2 Member 4), also upregulated in the TBT group. *Slc2a4* is a member of the solute transporter 2 (facilitated glucose transporter) family and encodes the main glucose transporter present in skeletal and cardiac muscles and adipose tissue, *GLUT4*. Expression of *Slc2a4*/*GLUT4* is majorly involved in glucose removal from tissues and, consequently, in glycemic homeostasis, playing an important role in the pathophysiology of diseases such as Type 1 and 2 Diabetes Mellitus and Obesity [26]. Downregulation of GLUT4 in obesity is an important factor contributing to impaired insulin-stimulated glucose transport in adipocytes [27]. As reviewed by Yohannes Tsegyie Wondmkun [28], defective insulin receptor signaling is a major component of obesity-associated insulin resistance in humans. Bazhan et al. [29] reported that levels of the gene responsible for glucose uptake in white adipose tissue in mice, *Slc2a4*, were subject to age-related changes, with *Slc2a4* expression increasing from young age to early adulthood and decreasing with age from adulthood onwards. Progression from early to late adulthood is commonly accompanied by an impaired glucose metabolism, including increased plasma insulin levels and impaired glucose tolerance. In agreement, Carvalho et al. [30] showed, also in mice, that reduced expression of *Slc2a4* in white adipose tissue is associated with the development of impaired glucose tolerance and insulin resistance, while its overexpression is linked to insulin sensitivity. Insulin stimulates the transport of glucose and the synthesis of triglycerides (lipogenesis), in addition to inhibiting lipolysis, which may be responsible for excessive accumulation of adipose tissue. Thus, insulin resistance in obesity is exhibited by reduced insulin-stimulated glucose transport and metabolism in adipocytes, and by impaired suppression of hepatic glucose production [31]. Kamstra et al. [32] showed in 3T3-L1 cells, that exposure to endocrine disruptors (e.g. EDC BDE-47) increases the expression of specific adipogenesis markers such as *Slc2a4*, through activation of peroxisome proliferator-activated receptor (PPARγ). We observed that the expression of *Slc2a4*, a differentiated adipocyte marker gene [33], is potentially regulated by lncRNA Gm6277, and its high expression can be explained by exposure to endocrine disruptors, as described above. Endocrine disrupting chemicals promote adipogenesis by altering fat cell development and/or increasing energy storage in adipose tissue [34], which, in turn, can be inherited by subsequent non-exposed generations, as demonstrated by Chamorro-Garcia et al. [35]. We suggest that regulation of lncRNAs and their gene-partners in the white gonadal adipose tissue of mice ancestrally exposed to the EDC TBT may be related to the control of adipogenesis, suggesting that this regulation may be epigenetically inherited.

The second lncRNA with the highest number of mRNA targets, the TBT-downregulated Gm10804. Recently, Li and colleagues [36] reported that low expression of Gm10804 improves glucose and lipid metabolism disorders in hepatocytes from mice exposed to high glucose, which is of importance as the liver plays a key role in adjusting glucose levels, in turn affecting energy homeostasis in other tissues [37]. One of the correlated target mRNAs of this lncRNA is the TBT-downregulated *Slc27a2* mRNA, also known as *FATP2*. *Slc27a2* plays a key role in lipid metabolism through fatty acid transport and/or activation of very long-chain fatty acids and is linked with activation and/or inhibition of the transcription factors PPARγ (in adipocytes), PPARα (liver) and PPARβ (adipocytes), regulating the expression of several genes involved in lipid metabolism [38]. Further, Choi and colleagues [39] reported in C57BL/6 J mice, that reduced expression of genes involved in lipolysis and uptake and transport of fatty acids (such as *Slc27a2*) in response to a high-fat diet (HFD) can reduce β-oxidation, resulting in excessive fat accumulation. Chamorro-Garcia et al. [35] have previously demonstrated that exposure of pregnant F0 mice to TBT led to transgenerational effects on the accumulation of lipids in white adipose tissue and liver, and to the increase in expression of hepatic genes involved in the storage/transport of lipids, in all future generations evaluated. Early exposure to endocrine-disrupting chemicals may alter metabolic homeostasis points, predisposing exposed individuals and their offspring to store more fat [40]. Here in our study, we used the RNA-Seq data produced by Chamorro-Garcia et al. [1], from gonadal adipose tissue samples from F4 mice transgenerationally exposed to EDC TBT in F0, which were subjected to a high fat diet challenge (HDF– 21.2% Kcal from fat) for 6 weeks, to assess the interaction between EDC TBT and fat accumulation. Our results suggest that low expression and the positive correlation of the mRNA *Slc27a2* with the lncRNA Gm10804 in the gonadal white adipose tissue of mice ancestrally exposed to the obesogenic substance TBT, may alter lipogenic and lipolytic pathways, reflecting in increased fat storage as well as decreased fat mobilization, as observed in these mice. This corroborates previous reports of association of lncRNAs with several metabolic conditions such as obesity, type 1 diabetes mellitus, type 2 diabetes mellitus and non-alcoholic fatty liver disease [41].

LncRNA Rian, which is orthologous with the lncRNA MEG8 in humans, was down-regulated in our study. One of the correlated target mRNAs of this lncRNA is the TBT-downregulated Inhba, also known as activin A. Activin A is a secreted adipokine composed of two subunits of inhibin βA (INHBA) and is highly expressed in the adipose tissue of obese patients when compared to lean individuals. INHBA is a member of the transforming growth factor-β superfamily and regulates a number of cellular events, including regulation of cancer cell growth and metastasis, apoptosis and, primarily, proliferation and differentiation of human embryonic stem cells. Zaragosi and colleagues [42] analyzed the transcriptome of human adipose tissue-derived stem cells (hMADS) and identified that activin A is expressed in adipose progenitors of various human fat depots and is dramatically downregulated as these progenitor cells undergo adipogenesis. Thus, we suggest that downregulation of mRNA *Inhba*, positively correlated with the lncRNA Rian, may be associated to excessive accumulation of adipose tissue resulting from exposure to the endocrine disruptor tributyltin and adipogenic pathways.

Evidence shows that in many complex diseases (e.g. cancer, obesity, diabetes), expression levels of lncRNAs and mRNAs can be significantly altered through DNA methylation, which plays a vital role as an epigenetic regulator [43]. Hernando-Herraez et al. [44] reported that methylation of lncRNA promoters is involved in a variety of biological processes and can lead to silencing or activating their expression. Dysregulation of lncRNA expression, by means of promoter methylation, can directly affect expression of their target mRNAs, or indirectly affect mRNAs controlled by miRNAs that are targets of competing endogenous lncRNAs [45]. Methylation in the promoter region of genes and lncRNAs is a major component of epigenetic regulation, however much less is known about DNA methylation outside of proximal promoters [46]. Albeit less investigated, methylation of regulatory regions outside of promoters are also able to regulate gene expression, as reviewed by Ordoñez and contributors [47]. Chamorro-Garcia et al. [1] analyzed differentially methylated regions (DMRs) and isoDMB regions (genomic regions containing differentially methylated DNA blocks with similar methylation profile) and associated to differentially expressed genes related to metabolism. Using their methylation data, we found DE lncRNAs and mRNAs in regions classified by the authors as regions II and III (Chamorro-Garcia et al., [1]), with 11 lncRNAs and 183 mRNAs that were downregulated by TBT located in hypermethylated regions, and 9 lncRNAs and 58 mRNAs that were TBT-upregulated located in hypomethylated regions. Similar to the findings of Chamorro-Garcia et al. [1], these target mRNAs encode proteins that participate in pathways involved in fatty acid metabolism, such as β-oxidation, citric acid cycle and glycolysis, such as the *Slc27a4* mRNA. Our results support and extend the findings of Chamorro-Garcia et al. [1] and suggest that some of the altered expression profiles of mRNAs and lncRNAs, observed transgenerationally following exposure to TBT, could be directly related and partly explained by alterations in methylation profile.

## Conclusions

Our analyses showed that ancestral exposure to obesogenic substances seems to play an important role in the low and/or high expression of mRNAs, potentially regulated by lncRNAs, which act in the glucose and lipid metabolism pathways, which are directly related to the adipogenesis process. Therefore, further studies are needed on the molecular and biological roles of lncRNAs as potential regulators of white adipose tissue functions.

## Supporting information

S1 Table (A and B) lncRNAs differentially expressed in contrast to EDC TBT and DMSO control groups. (XLSX)

S2 Table Interactions between DE lncRNA and mRNA (Pearson`s correlation r ≥ |0.80|; p-value <0.05). (XLSX)

S3 Table lncRNA-mRNA interactions presented significant binding potential (normalized deltaG analysis in LncTar tool (ndG < -0.10)). (XLSX)

S4 Table Results of cis lncRNAs-mRNAs pair prediction analysis (performed in the FEELnc tool). (XLSX)

S5 Table KEGGs pathways of the top 5 differentially expressed lncRNAs based on their mRNA interaction. (XLSX)
